# Ventricular Tachycardia Recurrence After Catheter Ablation in Nonischemic Cardiomyopathy with Mildly to Moderately Reduced Ejection Fraction

**DOI:** 10.1016/j.cjco.2026.04.009

**Published:** 2026-05-01

**Authors:** Boldizsar Kovacs, Leon Dinshaw, Alvaro Marco del Castillo, Christiane Jungen, Stefan Kurath-Koller, Stefan Stojković, Francesco Santoro, Giacomo Mugnai, Laura Perrotta, Rachel M.A. ter Bekke, Kevin Vernooy, Paul G.A. Volders, Bert Vandenberk

**Affiliations:** a Department of Cardiology, Inselspital Bern, University Bern, Berne, Switzerland; b Department of Cardiology, Sana Hanse Hospital Wismar, Wismar, Germany; c Hospital Universitario 12 de Octubre, Madrid, Spain; d Department of Cardiology and Vascular Medicine, West German Heart and Vascular Center, University Hospital Essen, Essen, Germany; e Division of Pediatric Cardiology, Department of Pediatrics, Medical University Graz, Graz, Austria; f University Clinic for Internal Medicine II, Divison of Cardiology, Medical University of Vienna, Vienna, Austria; g Cardiology Unit, Department of Medical and Surgery Sciences, University of Foggia, Foggia, Italy; h Division of Cardiology, Cardio-Thoracic Department, University Hospital of Verona, Verona, Italy; i Arrhythmia Unit, Cardio-thoracic Department, Careggi University Hospital, Florence, Italy; j Department of Cardiology, Maastricht University Medical Center +, Cardiovascular Research Institute, Maastricht, the Netherlands; k Department of Cardiovascular Sciences, KU Leuven, Leuven, Belgium

**Keywords:** ventricular tachycardia, catheter ablation, non-ischemic cardiomyopathy, moderately reduced ejection fraction, preserved ejection fraction, left ventricular ejection fraction, systematic review

## Abstract

**Background:**

Patients with nonischemic cardiomyopathy (NICM) are at risk for sustained ventricular tachycardia (VT). Implantable cardioverter-defibrillators (ICDs) are guideline-recommended for secondary prevention, but the necessity of routine ICD implantation after successful catheter ablation (CA) in patients with preserved or mildly reduced left ventricular ejection fraction (LVEF > 35%) remains uncertain. This review summarizes the current evidence.

**Methods:**

A PROSPERO-registered systematic review was conducted according to Preferred Reporting Items for Systematic Reviews and Meta-Analyses (PRISMA) guidelines. PubMed, Embase, and Cochrane were searched through June 2025. Studies including adults with NICM and LVEF > 35% undergoing CA for sustained monomorphic VT were eligible. Data on patient characteristics, ablation outcomes, VT recurrence, mortality, and ICD therapies were extracted. Results were descriptively synthesized, and weighted means were calculated when feasible.

**Results:**

Eleven studies (1995-2024), including 1430 patients, were identified; 936 (66%) were analyzable. The weighted mean LVEF was 38% ± 14%; mean age was 59 ± 14 years. Sustained VT recurrence ranged from 14%-67% during a weighted mean follow-up period of 608 ± 745 days. Procedural noninducibility consistently predicted lower recurrence rates (18%-32%), than those in inducible patients (up to 75%). Two studies using cardiac magnetic resonance imaging found non-circumferential mid-myocardial late gadolinium enhancement with septal involvement associated with higher recurrence risk. Risk of bias was moderate to serious.

**Conclusions:**

In NICM patients with LVEF > 35%, noninducibility after CA predicts favourable arrhythmic outcomes. Non-circumferential mid-myocardial late gadolinium enhancement with septal involvement, and inducibility after VT ablation, identify higher-risk patients.

**Registration:**

PROSPERO CRD420251075109.

## Central Illustration

**Figure:**
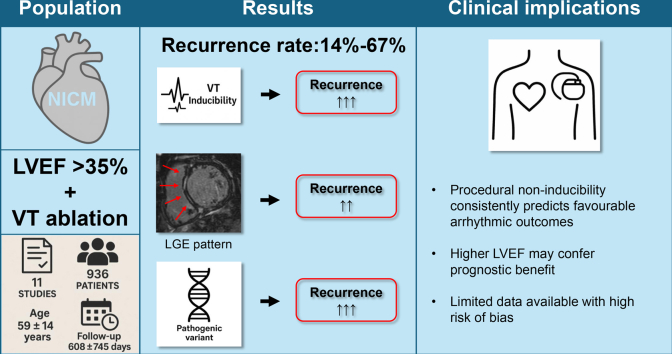

## Résumé

**Contexte:**

Les patients atteints de cardiomyopathie non ischémique (CNI) présentent un risque de tachycardie ventriculaire (TV) soutenue. Les défibrillateurs cardioverteurs implantables (DCI) sont recommandés par les lignes directrices en prévention secondaire, mais la nécessité d'une implantation systématique de DCI après une ablation par cathéter (AC) réussie chez les patients présentant une fraction d’éjection ventriculaire gauche (FEVG) préservée ou légèrement réduite (FEVG > 35 %) reste incertaine. Cette revue de littérature résume les données actuelles.

**Méthodologie:**

Une revue systématique enregistrée dans PROSPERO a été menée conformément aux lignes directrices PRISMA. Des recherches ont été effectuées dans PubMed, Embase et Cochrane jusqu'en juin 2025. Les études incluant des adultes atteints de CNI et présentant une FEVG > 35 % et ayant bénéficié d'une AC pour une TV monomorphe soutenue étaient éligibles. Les données relatives aux caractéristiques des patients, aux résultats de l'ablation, à la récidive de la TV, à la mortalité et aux thérapies par DCI ont été extraites. Les résultats ont été synthétisés de manière descriptive et des moyennes pondérées ont été calculées lorsque cela était possible.

**Résultats:**

Onze études (1995-2024) portant sur 1 430 patients ont été identifiées ; 936 (66 %) étaient analysables. La FEVG moyenne pondérée était de 38 ± 14 %, l'âge moyen de 59 ± 14 ans. La récidive de TV soutenue variait de 14 à 67 % au cours d'un suivi moyen pondéré de 608 ± 745 jours. L'absence d'inductibilité lors de l'intervention prédisait systématiquement des taux de récidive plus faibles (18–32 %) par rapport aux patients inductibles (jusqu'à 75 %). Deux études utilisant l'imagerie par résonance magnétique cardiaque ont mis en évidence qu'un rehaussement tardif au gadolinium (RTG) non circonférentiel du myocarde moyen avec atteinte septale était associé à un risque de récidive plus élevé. Le risque de biais était modéré à grave.

**Conclusions:**

Chez les patients atteints de CNI présentant une FEVG > 35 %, la non-inductibilité après AC prédit une évolution rythmique favorable. Un rehaussement tardif au gadolinium (RTG) non circonférentiel au niveau du myocarde moyen avec atteinte septale et la présence de patients inductibles après ablation de la TV permettent d'identifier les patients à plus haut risque.

Ventricular tachycardia (VT) is a potentially life-threatening arrhythmia, particularly in patients with structural heart disease. Patients with nonischemic cardiomyopathy (NICM) are at risk for sustained VT, with an event rate of approximately 4.5% per year, depending on the underlying disease, the left ventricular ejection fraction (LVEF), and late gadolinium enhancement (LGE) on cardiac magnetic resonance imaging (CMR).^1^ Implantable cardioverter-defibrillators (ICDs) have been shown to reduce the risk of sudden cardiac death in patients with NICM and VT in the secondary prevention setting. Accordingly, current guidelines strongly recommend ICD implantation in patients with structural heart disease (including NICM) who have experienced sustained VT in the absence of a reversible cause.^2^^,^^3^

Catheter ablation (CA) in ICD carriers significantly reduces the arrhythmia burden and the frequency of ICD therapies, and it is more effective than antiarrhythmic drugs.^4^, ^5^, ^6^, ^7^, ^8^ Emerging data suggest that patients with an LVEF > 35% and ischemic cardiomyopathy and hemodynamically tolerated VT may experience favourable long-term outcomes after successful VT ablation, even in the absence of ICD implantation.^9^, ^10^, ^11^, ^12^, ^13^

The NICM population generally is more heterogeneous than the population with ischemic cardiomyopathy (ICM), encompassing multiple aetiologies, and the arrhythmogenic substrate is less well characterized. In addition, studies on CA of monomorphic VT in patients with mixed forms of NICM have demonstrated a lower rate of acute and long-term VT recurrence, compared to the rate in those with ischemic cardiomyopathy.^14^, ^15^, ^16^ However, these studies included primarily patients with severely reduced LVEF. Given this context, the VT recurrence rate after ablation in NICM and the benefit of ICD implantation following successful CA in patients with NICM and preserved or mildly reduced LVEF (> 35%) remains uncertain.

The aim of this systematic review is to address this critical knowledge gap by evaluating the arrhythmic outcomes of patients with NICM and LVEF > 35% who have undergone successful CA for monomorphic VT.

## Methods

The search strategy followed the best practice guidelines from the Preferred Reporting Items for Systematic Reviews and Meta-Analyses (PRISMA) statement.^17^^,^^18^ The protocol for this systematic review has been registered in the international prospective register for systematic reviews (PROSPERO) on June 22, 2025 (registration number: CRD420251075109). Institutional review board approval was not required.

### Data sources and search strategy

A comprehensive literature search was performed in the PubMed, Embase, and Cochrane databases for the period from their inception through June 22, 2025. The search combined relevant subject headings and free-text terms related to “non-ischemic cardiomyopathies” (eg, myocarditis, dilated cardiomyopathy, arrhythmogenic cardiomyopathy), “catheter ablation,” and “ventricular tachycardia.” The detailed search strings for each database are provided in Supplemental Appendix S1. Only studies published in English were considered. Reference lists of included studies were screened to identify additional relevant publications.

### Study selection and eligibility criteria

Search results were imported into Rayyan (Cambridge MA), a Web-based screening platform that allows blinded and independent assessment by multiple reviewers.^19^ After uploading the results of the searches, duplicates were removed semi-automatically. Subsequently, 4 investigators (B.K., A.M.C., L.D., and B.V.) independently screened titles and abstracts. Eligible studies were original research articles reporting on adult patients with NICM and study-level mean LVEF > 35%, undergoing CA for sustained monomorphic VT. Given the lack of randomized controlled trials, both retrospective and prospective observational studies reporting outcomes after CA for sustained monomorphic VT in patients with NICM and a mean LVEF > 35% were included. Studies including only patients with hypertrophic cardiomyopathy (HCM), arrhythmogenic right or left ventricular cardiomyopathy (ARVC), or cardiac sarcoidosis (CS) were excluded a priori. These disease entities are characterized by distinct arrhythmogenic substrates, natural history, and risk stratification strategies that are not determined primarily by LVEF. CS is additionally limited by heterogeneous diagnostic criteria and fluctuating inflammatory activity, whereas ARVC and HCM are genetically mediated cardiomyopathies with extensive disease-specific outcome data.^20^^,^^21^ Inclusion of these subtypes would have limited interpretability with respect to the study population “NICM.” In the case of mixed cohorts, the subgroup with only NICM was considered for analysis. Conference abstracts reporting required data were considered eligible. Studies were excluded if any of the following were true: they reported mixed populations without a separate analysis of the relevant subgroup; essential outcome data were missing; the population was not clearly defined; or double-counting was suspected in the case of multiple publications by the same research group. Case reports, case series, and review articles were excluded.

**Figure 1:**
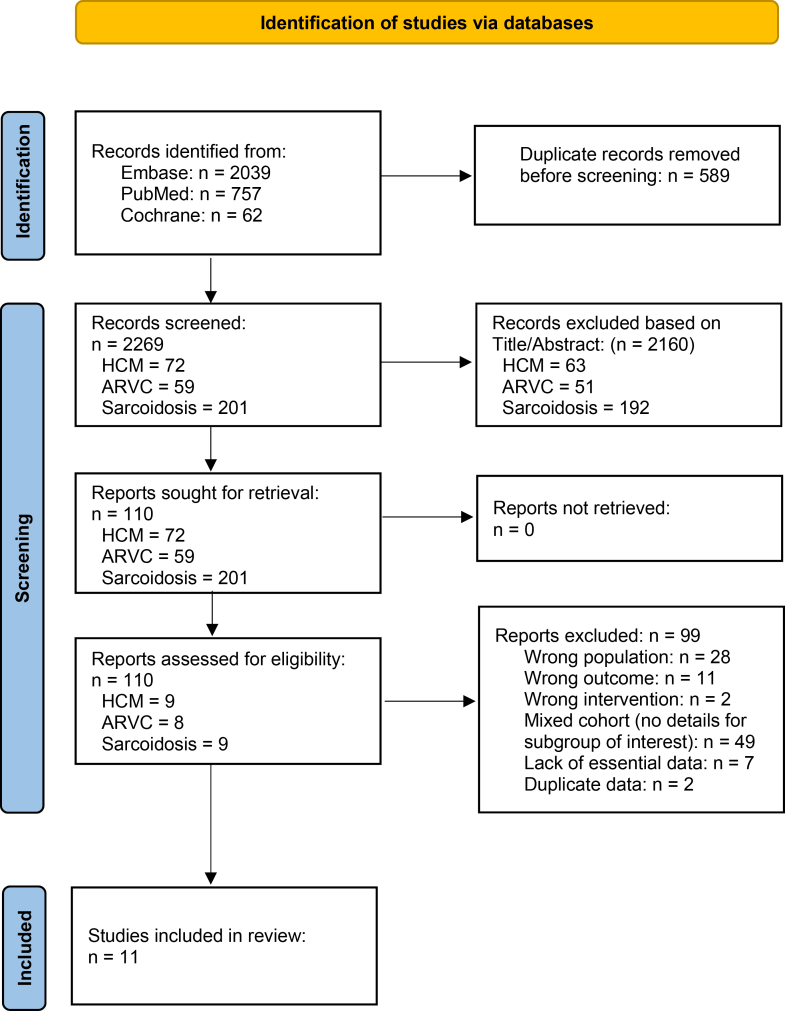
Study flowchart. ARVC, arrhythmogenic right or left ventricular cardiomyopathy; HCM, hypertrophic cardiomyopathy. Source: Page MJ, et al. BMJ 2021;372:n71. doi: 10.1136/bmj.n71. This work is licensed under CC BY 4.0. To view a copy of this license, visit https://creativecommons.org/licenses/by/4.0/.

After initial blinded screening, the results were unblinded once each study was reviewed by at least 2 investigators, and discrepancies were resolved by consensus between the investigators. Subsequently, full-text manuscripts were assessed to validate the study design, patient demographics, and outcomes. The full-text assessment was subsequently performed independently in pairs, followed by discussion until consensus. Studies from the same research group were carefully reviewed to avoid duplicate data and ensure inclusion of the cohort that was most complete or had the longest follow-up period. The PRISMA flowchart summarizing the selection process is presented in Figure 1.

### Data extraction

Data extraction was performed in pairs using a predefined data extraction template. Each pair independently extracted data, followed by discussion and resolution of discrepancies through consensus. A third reviewer (B.V.) was available for adjudication as needed.

Extracted data included the following: (i) study characteristics (first author, year, country, study design); (ii) population details (sample size, NICM subtype, baseline LVEF, LGE on CMR, antiarrhythmic drug therapy); and (iii) outcomes (VT-inducibility after index ablation, VT recurrence, all-cause and cardiovascular mortality, appropriate ICD therapy or shocks, and heart transplantation during the follow-up period).

### Quality assessment

The Risk of Bias in Non-randomized Studies of Interventions (ROBINS-I) tool was used to evaluate study quality.^22^ Each study was assessed independently by 2 reviewers (A.M.C., C.J.), with a third reviewer (B.K.) available to resolve disagreements. The ROBINS-I tool evaluates bias across 7 domains, including confounding, selection, intervention classification, deviations from intended interventions, missing data, outcome measurement, and reporting.^22^

### Data synthesis and statistical analysis

Due to the heterogeneity of studies and endpoints, the reported data were not suitable for a meta-analysis. Data were collected as numbers (%), mean ± standard deviation (SD). If not available, mean ± SD were estimated.^23^ When feasible, weighted values were calculated if the median and interquartile range or range values were inconsistently available.

## Results

A total of 11 studies were included in this systematic review, comprising a total of 1430 patients with NICM undergoing catheter ablation for VT. Studies were published between 1995 and 2024. The selected studies comprised 9 retrospective cohort studies and 2 prospective studies, with sample sizes ranging from 7 to 780 patients (Table 1). The weighted mean LVEF of all included patients was 38% ± 14%.

**Table 1:** Publication characteristics

Study	Journal	Country	Study type	Total study population	CMP	LVEF, %	Key inclusion criteria	Key exclusion criteria
Kottkamp et al.^27^ (1995)	Circulation	GER	P	8	DCM	30 ± 9	Systolic impairment	CAD, VHD, CHD
Delacretaz et al.^28^ (2000)	JCE	US	R	26	DCM	38 ± 14	Sustained monomorphic VT	CAD, VHD, CHD, ACM
Piers et al.^30^ (2013)	Circ AE	US	R	45	DCM	44 ± 14	Systolic impairment + scar on CMR/ mapping	CAD, CHD, VHD, HCM, ACM, LVNC, MYO, CS, toxic / tachy CMP
Sanzo^31^ (2013)∗	Europace	ITA	R	7	MYO	44 ± 6	Post-MYO + VT + ablation	NR
Oloriz et al.^33^ (2014)	Circ AE	ITA	R	87	NICM	37 ± 13†	NICM + VT + ablation	CAD, ARVC, CS, HCM, LVNC
Muser et al.^26^ (2016)	Circ AE	US	R	282‡	DCM	36 ± 13	LVEF < 50% despite OMT	CAD, CHD, VHD, HCM, ACM, LVNC, MYO, CS, toxic / tachy CMP
Vaseghi et al.^15^ (2018)	JACC EP	US/ITA	R	780	NICM	37 ± 13	NICM + VT + ablation	Mixed ICM+NICM
Nishimura et al.^25^ (2021)	Heart Rhythm	US	R	25	NICM	37 ± 17†	Non-idiopathic anteroseptal/peri-aortic VT	NR
Bennett et al.^32^ (2022)	Heart Rhythm	US	P	77	DCM	40 ± 14	Mildly reduced LVEF, VT ablation	ICM, LVEF < 45%
Kovacs et al.^24^ (2023)	JCE	US	R	43	NICM	45 ± 16	Left-sided NICM	ICM
Li et al.^29^ (2024)	JACC:Asia	CHN	R	50	MYO	52 ± 13	MYO + VT	CAD, GCM, CS, no biopsy

ACM, arrhythmogenic cardiomyopathy; CAD, coronary artery disease; CHD, congenital heart disease; CHN, China; Circ AE, *Circulation: Arrhythmia and Electrophysiology*; CMP, cardiomyopathy; CMR, cardiac magnetic resonance imaging; CS, cardiac sarcoidosis; DCM, dilatative cardiomyopathy; GCM, giant cell myocarditis; GER, Germany; HCM, hypertrophic cardiomyopathy; ICM = ischemic cardiomyopathy; ITA, Italy; JACC, *Journal of the American College of Cardiology;* JACC-EP, *JACC: Clinical Electrophysiology*; JCE, *Journal of Cardiovascular Electrophysiology*; LVEF, left ventricular ejection fraction; LVNC, left ventricular noncompaction; MYO, myocarditis; NICM, nonischemic cardiomyopathy; NR, not reported; OMT, optimal medical treatment, P, prospective; R, retrospective; VHD, valvular heart disease; VT, ventricular tachycardia;.

∗ Conference abstract.

† The mean ± standard deviation was calculated from the mean and range or IQR.

‡ The supplementary material of the study reports a total study population of 348 patients.

### Patient populations and study characteristics

The study population considered for this review consisted of 936 patients (65% of the total population); the remaining patients were excluded based on the exclusion criteria. The largest study reviewed included 66% of the total review study population.^15^ All patients had NICM and had undergone CA for VT. The most common underlying heart disease was nonischemic dilatative cardiomyopathy (DCM), followed by myocarditis and valvular heart disease (VHD). One study reported the genetic work-up of all included patients, and one reported on selected patients with lamin *A/C* cardiomyopathy.^24^^,^^25^ The weighted mean LVEF of the review study population was 37% ± 15%, and the weighted mean age was 59 ± 14 years. Where reported, the proportion of patients in New York Heart Association class III-IV ranged from 0% to 33%. Most included patients were implanted with an ICD, and class-III antiarrhythmic drug use in the studies ranged from 20% to 100%. Four studies reported on a history syncope or hemodynamic tolerability of VT prior to the procedure.^26^, ^27^, ^28^, ^29^ Of the total of 11 studies, 8 defined a successful ablation procedure as VT-noninducibility (with noninducibility variably defined, ranging from absence of clinical VT to absence of any inducible VT, using heterogeneous programmed stimulation protocols); the remaining 3 defined it as clinical VT noninducibility or late potential / local abnormal ventricular activation abolition in addition to VT noninducibility (Table 2).

**Table 2:** Baseline characteristics of study population

Study	Review study population	Age, Y	LVEF of review study population, %	NYHA class III & IV	ICD	AAD class III	Ablation success definition
Kottkamp et al.^27^ (1995)	3	49 ± 5	40 ± 3	NR	3 of 3 (100)	3 of 3 (100)	Clinical VT NI
Delacretaz et al.^28^ (2000)	10	52 ± 17	48 ± 10	NR	6 of 10 (60)	2 of 10 (20)	VT NI
Piers et al.^30^ (2013)	45	60 ± 16	44 ± 14	15 of 45 (33)	45 of 45 (100)	22 of 45 (49)	VT NI
Sanzo∗^,^^31^ (2013)	7	50 ± 17	44 ± 6	NR	3 of 7 (43)	NR	VT NI
Oloriz et al.^33^ (2014)	28	53 ± 6§	57 ± 7†	0 of 28 (0)	11 of 28 (39)	NR	VT NI and LP abolition
Muser et al.^26^ (2016)‡	145	59 ± 15†	NR	84 of 282 (30)	240 of 282 (85)	166 of 282 (59)‖	VT NI
Vaseghi et al.^15^ (2018)	DCM 518	60 ± 13	33 ± 13	34 of 518 (7)	588 of 780 (80)	NR	VT NI
	VHD 50	65 ± 11	31 ± 13	30 of 50 (60)			
	MYO 50	50 ± 15	43 ± 16	16 of 50 (32)			
Nishimura et al.^25^ (2021)	10∗∗^,^¶	66 ± 7	43 ± 12†	NR	9 of 10 (90)	6 of 10 (60)	LAVA abolition and VT NI
Bennett et al.^32^ (2022)	20	61 ± 15	54 ± 5	NR	16 of 20 (80)	8 of 20 (40)	VT NI
Kovacs et al.^24^ (2023)	27	57 ± 14	48 ± 15	NR	27 of 27 (100)	13 of 27 (50)	VT NI
Li et al.^29^ (2024)	23	43 ± 13	55 ± 10	NR	4 of 23 (17)	23 of 23 (100)	VT NI

The values are n (%) or mean ± standard deviation unless otherwise stated. The N values are the review study population or the total study population, as indicated.

AAD, anti-arrhythmic drug; DCM, dilatative cardiomyopathy; ICD, implantable cardioverter defibrillator; LP, late potential; LAVA, local abnormal ventricular activities; LMNA, Lamin A/C; LVEF, left ventricular ejection fraction; MYO, myocarditis; NI, noninducibility; NR, not reported;.Ref, reference, VHD, valvular heart disease; VT, ventricular tachycardia.

∗ Conference abstract.

† The mean ± standard deviation was calculated from the mean and range or interquartile range.

‡ 145 of 282 patients (51%) had an LVEF > 35%; however, the supplementary materials report on a total of 164 patients. No differential reporting for baseline variables based on LVEF available; the percentages refer to the total study population.

§ The mean refers to the total study population.

‖ A small additional percentage of patients took sotalol, but the number is not reported.

¶ The subgroup with partial septal late gadolinium enhancement and higher LVEF in the study.

∗∗ One patient was LMNA positive.

### Outcomes

#### Sustained VT recurrence and ICD therapies

Follow-up durations varied widely, from a mean of 223 ± 77 days to 1461 ± 1083 days, with a weighted mean follow-up of 608 ± 745 days. All studies reported the sustained VT recurrence rate, which ranged from 14% to 67%. Appropriate ICD therapy and shock rates were reported in only 3 studies (Table 3). The highest recurrence rate was reported in a small retrospective study from 1995 in which two thirds of the patients had sustained VT recurrence and ICD therapies.^27^ Of note, in this study, 6 of 8 patients had inducible VTs at the end of the procedure. In another study with a VT recurrence rate of 21%, patients with an LVEF > 35% or ≤ 35% were analyzed separately. Those in the higher LVEF category had a recurrence-free survival rate of 78%, compared to those with a lower LVEF, who had 60% recurrence-free survival.^26^

**Table 3:** Outcomes

Study	Follow-up, d	All-cause mortality	Cardiovascular mortality	Sudden cardiac death	VT Non-inducibility	Sustained VT recurrence	Appropriate ICD therapy	Appropriate ICD shock	Heart transplant
Kottkamp et al.^27^ (1995)	223 ± 77	NR	NR	NR	1 of 3 (33)	2 of 3 (67)	2 of 3 (67)	2 of 3 (67)	0 of 0
Delacretaz et al.^28^ (2000)	444 ± 347	NR	NR	NR	8 of 10 (80)	2 of 10 (20)	NR	NR	NR
Piers et al.^30^ (2013)	731 ± 564¶	6 of 45 (13)	5 of 45 (11)	0 (0)	17 of 45 (38)	24 of 45 (53)§	NR	20 of 45 (44)	2 of 45 (4)
Sanzo^31^ (2013)∗	731‖	NR	NR	NR	5 of 7 (71)	1 of 7 (14)	NR	NR	NR
Oloriz et al.^33^ (2014)	591 ± 172¶	0 of 28 (0)	0 of 28 (0)	0 of 28 (0)	4 of 28 (14)	8 of 28 (29)	3 of 28 (11)	2 of 28 (7)	0 of 28
Muser et al.^26^ (2016)	1461 ± 1083	43 of 282 (15)‡	NR	0 of 282 (0)	216 of 262 (82)∗∗	58 of 282 (21)‡	NR	NR	24 of 282 (9)‡
Vaseghi et al.^15^ (2018)	390 ± 497†^,^¶	137 of 780 (18)	NR	NR	280 of 518 (54)	DCM 166 of 518 (32)	NR	NR	47 of 780 (6)
					20 of 50 (40)	VHD 22 of 50 (43)			
					31 of 50 (62)	MYO 22 of 50 (23)			
Nishimura et al.^25^ (2021)	436 ± 384†^,^¶	0 of 10 (0)	0 of 10 (0)	0 of 10 (0)	9 of 10 (90)	8 of 10 (80)	NR	NR	4 of 25 (16)†
Bennett et al.^32^ (2022)	526 ± 203¶	0 of 20 (0)	0 of 20 (0)	0 of 20 (0)	11 of 20 (55)	13 of 20 (35)	NR††	NR††	0 of 20 (0)
Kovacs et al.^24^ (2023)	1242 ± 1060†	2 of 43 (5)†	1 of 43 (2)†	NR	33 of 43 (77)†	4 of 26 (15)	NR	NR	2 of 43 (5)†
Li et al.^29^ (2024)	338 ± 93†^,^¶	0 of 23 (0)	0 of 23 (0)	0 of 23 (0)	15 of 23 (65)	8 of 23 (35)	NR	NR	0 of 23 (0)

The values are n (%) unless otherwise stated. The N values are the review study population or the total study population, as indicated.

DCM, dilatative cardiomyopathy; ICD, implantable cardioverter defibrillator; LVEF, left ventricular ejection fraction; MYO, myocarditis; NR, not reported; Ref, reference; VHD, valvular heart disease; VT, ventricular tachycardia.

∗ Conference abstract.

† Refers to the total study population.

‡ The sustained VT recurrence-free rate in the LVEF > 35% subgroup at the end of the follow-up period was 77%-78%; the death/transplant-free survival was 88%-90%.

§ The recurrence rate for patients with procedural success was 3 of 17 (18%).

‖ No standard deviation was reported in the article.

¶ The mean ± standard deviation was calculated from the mean and range or interquartile range (IQR).

∗∗ Programmed stimulation was performed in 262 of 282 patients; the success rate after multiple procedures was reported.

†† Median appropriate ICD therapies and shocks during follow-up were 0 [IQR 0-1.5] and 0 [IQR 0-0], respectively.

#### Noninducibility and sustained VT recurrence

Four studies reported the VT recurrence rate in a subgroup of patients with VT noninducibility at the end of the index CA procedure. Piers et al. found a recurrence rate of 18% in noninducible patients, compared to 75% in the still-inducible patients, confirming that inducibility at the end of the procedure is a significant predictor of VT recurrence during the follow-up period.^30^ Noninducibility was achieved in 33% and 79% of patients, and none of these had any VT recurrence during the follow-up period in Kottkamp et al and Delacretaz et al, respectively.^27^^,^^28^ In the study reported by Sanzo et al., recurrence occurred in 1 of 2 patients with only “partial” acute procedural success.^31^

Vaseghi et al. investigated the VT recurrence rate according to NICM subtype.^15^ The subgroups of patients with higher rates of noninducibility at the end of the index procedure (myocarditis 76%, DCM 63%, and VHD 44%) had lower VT recurrence rates during the follow-up period (myocarditis 23%, DCM 32%, and VHD 43%), although the impact of noninducibility was not separately investigated.^15^ Muser et al. did not provide separate recurrence rates for noninducible patients; however, they identified noninducibility alongside LVEF ≤ 35% as the only significant predictors of reduced VT recurrence.^26^ Similarly, Bennett et al. did not report on the impact of noninducibility on the recurrence rate; however, the subgroup with LVEF > 35% had a higher rate of noninducibility at the end of the index CA procedure (44% vs 25%), but no difference in the rate of VT recurrence during the follow-up period (65% vs 63%).^32^ Oloriz et al. identified noninducibility as a predictor for VT recurrence during follow-up in only univariate, and not in multivariate, analysis.^33^ Further procedural characteristics are shown in Supplemental Table S1.

#### LGE on CMR and sustained VT recurrence

The prognostic effect of LGE on CMR was investigated in 2 studies. In the subgroup of patients with moderately reduced to preserved LVEF and no pathogenic variant, septal LGE was associated with VT recurrence after ablation. Specific patterns such as noncircumferential mid-myocardial LGE with septal involvement and the extent of LGE (scar burden) and scar depth were individually and independently associated with higher recurrence rates.^24^^,^^25^

#### Genotype and sustained VT recurrence

VT recurrence rate depending on genotype was reported in 2 studies. Kovacs et al. described a higher VT recurrence rate in patients with pathogenic variants (65%) vs in those with variants of unknown significance or negative genetic testing (15%).^24^ Nishimura et al. described only lamin A/C pathogenic variant–positive patients and found that two thirds had recurrence.^24^^,^^25^

#### Mortality and heart transplantation

All-cause mortality, when reported, ranged from 0% to 18% over the follow-up period. Cardiovascular mortality and sudden cardiac death were infrequently reported (Table 3). Among the studies reporting all-cause mortality, follow-up durations ranged from 338 ± 93 days^13^ to 1461 ± 1083 days.^26^ Mortality rates varied widely: some cohorts with longer follow-up periods reported relatively modest mortality (eg, Muser et al., 15% at ∼4 years^26^), whereas others with a shorter follow-up period reported higher rates (eg, the myocarditis subgroup in Vaseghi et al., 43% at ∼1 year^15^). Conversely, several studies with moderate follow-up period durations reported no deaths (Bennett et al.^32^; Li et al.^29^).

### Risk-of-bias assessment

Overall, the risk of bias across included studies was moderate to serious (Supplemental Table S2). Most studies were observational and were subject to confounding and selection biases, particularly due to the lack of randomization and incomplete adjustment for prognostic factors. The classification of interventions and deviations from intended protocols were also inconsistently reported. Measurement and reporting of outcomes often were not blinded, increasing the risk of detection bias.

## Discussion

The main findings of this systematic review investigating VT recurrence after CA in patients with NICM and LVEF > 35% undergoing CA for monomorphic VT are as follows: (i) procedural noninducibility remains the strongest and most consistent predictor of lower VT recurrence; (ii) LGE on CMR is associated with an increased VT recurrence rate, but data are very limited; and (iii) a higher LVEF may be associated with better arrhythmic outcomes, but this depends on the underlying heart disease (Central Illustration).

**Central Illustration:**
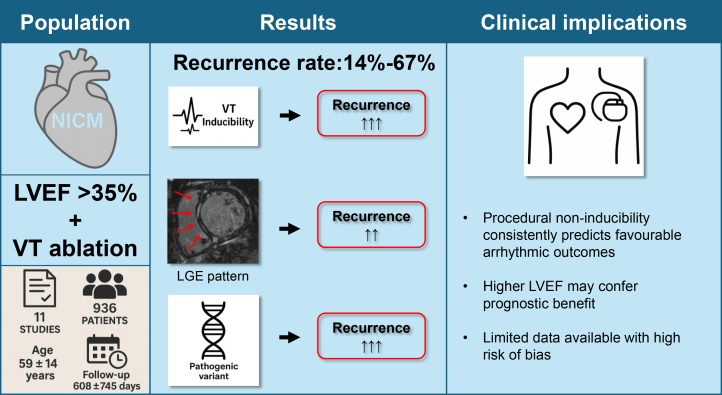
Ventricular tachycardia (VT) recurrence after VT-ablation in nonischemic cardiomyopathy (NICM) and left ventricular ejection fraction (LVEF) > 35%. Summary of the findings of the systematic review. LGE, late gadolinium enhancement.

A systematic review published in 2016 specifically evaluated whether noninducibility after VT-ablation procedures in patients with NICM is associated with an improved outcome. The investigators concluded that existing evidence points toward a lower VT recurrence and mortality rate in patients without VT-inducibility at the end of the ablation procedure, compared to the rate in patients with inducibility after VT ablation procedures.^34^ This study included patients with any subtype of NICM, in particular including those with ARVC, HCM, and CS. Although ablation outcomes for HCM and CS are often worse in available literature due to the difficult-to-treat substrate,^35^ patients with ARVC may have a more favourable outcome after VT ablation.^36^^,^^37^ Moreover, the majority of the included studies had a mean LVEF ≤ 35%, which then would indicate the necessity of ICD implantation even in a primary preventive setting.^3^ The studies reviewed in the above-mentioned meta-analysis with a mean LVEF > 35% and NICM were either probable duplicate studies within our study selection,^38^ or re-do VT ablation^39^ procedures.

In the present review, studies including NICM patients with well-studied phenotypes, either with known very good or very poor outcomes after VT ablation (eg, ARVC, CS, and lamin A/C, respectively) were excluded. A noteworthy study with a high reported sustained VT recurrence rate found a recurrence rate of 53% in patients with NICM and a mean LVEF of 44% ± 14%.^30^ What the genotype of these patients was is unclear, but they may represent a selected, more arrhythmogenic subgroup, which is supported by the relatively high mortality rate (13%) and very high ICD shock rate during the follow-up period (44%). Of note, in this study, the recurrence rate in patients with noninducibility at the end of the index CA procedure was considerably lower (18%), similar to rates in the other included studies. Studies investigating VT recurrence rate and mortality in patients with NICM who undergo VT ablation have consistently reported more favourable outcomes when VT noninducibility was achieved at the end of the procedure.^34^^,^^40^^,^^41^ However, noninducibility is a heterogeneous endpoint, and its prognostic value depends strongly on the extent of testing performed. Therefore, if noninducibility is to be used for risk stratification in this population, comprehensive and standardized stimulation protocols are mandatory to ensure an optimal risk–benefit balance.

Other studies with higher VT recurrence rates include Vaseghi et al, Li et al., and Bennett et al.^15^^,^^29^^,^^32^ In the first study, as expected, the higher recurrence rate was linked to lower LVEF and VT-inducibility at the end of the index CA procedure.^15^ In the second study, noninducibility was achieved in only 44%.^32^ Finally, the third study included patients with acute myocarditis undergoing VT ablation procedures, which is likely associated with worse outcomes and higher arrhythmia burden due to acute inflammation.^29^ Noninducibility is heterogeneous; the prognostic impact hinges on post-ablation stimulation rigour, which was insufficiently reported to confirm standardization across studies.

Across different NICM subtypes, LGE on CMR has consistently emerged as a strong and independent predictor of VT. In patients with DCM and preserved or moderately reduced LVEF, the presence of midwall LGE identified individuals at substantially higher risk of sudden cardiac death independent of ejection fraction.^42^ In a large multicentre series, LGE remained an independent predictor of ventricular arrhythmias and sudden death across ejection fraction ranges with epicardial, transmural, and combined septal plus free-wall patterns indicating particularly high risk.^43^^,^^44^ These studies however, did not investigate the VT recurrence rate after ablation in these populations. Only 2 of the included studies investigated the ability of CMR to help predict post-ablation recurrence. These studies found that a septal intramural LGE was a predictor for VT recurrence after ablation.^24^^,^^25^ Important to note is that both studies investigated patients with pathogenic variants as well (LMNA, TTN, etc.); however, these could be excluded from the analysis for this systematic review. As expected, patients with such variants had significantly higher rates of VT recurrence.^24^

The most current Canadian guidelines strongly recommend ICD implantation indication for patients with NICM and sustained VT.^2^ Whether secondary prophylactic ICD implantation should follow the one-size-fits all principle is an open question. Although this systematic review suggests a relatively low all-cause mortality rate following VT ablation in selected patients with NICM, the available evidence does not support any inference regarding the necessity or deferral of ICD implantation. The majority of patients in the included studies had ICDs in place, and both mortality and arrhythmic outcomes therefore are intrinsically influenced by device therapy. In addition, VT recurrence rates remained substantial across studies. Although patients rendered noninducible at the end of the ablation procedure appear to have more-favourable outcomes, this observation should be interpreted as hypothesis-generating only. Substrate characteristics, including the extent and location of LGE, as well as the presence of genetic cardiomyopathies, may further modify risk, even in patients with preserved or mildly reduced LVEF.^24^ Prospective studies with standardized arrhythmic endpoints, systematic genetic characterization, and continuous rhythm monitoring are required before any conclusions regarding post-ablation arrhythmic risk stratification in this population should be considered.^45^^,^^46^

### Limitations

Limitations of this study include the following: the heterogeneity of NICM etiologies; the variable reporting of both genetic cardiomyopathies and results of any genetic testing and New York Heart Association class; scarce reporting on the prognostic value of CMR; heterogeneous reporting of ICD therapies and mortality; inconsistent subgroup reporting for LVEF and noninducibility; and lack of systematic antiarrhythmic drug use reporting. Moreover, the range of the mean LVEF suggests that some included patients had an LVEF < 35%, limiting the specificity of the findings. Reporting of VT recurrence was inconsistent, and most studies did not clearly differentiate between clinically manifest VT recurrence and VT detected only through ICD interrogation. Retrospective designs, small sample sizes, lack of comparator groups, and the lack of randomized evidence further limit generalizability. No study was rated as being low risk across all domains, but the findings remain informative and should be interpreted cautiously. Future prospective studies with standardized methodologies are needed to strengthen the quality of evidence.

## Conclusions

In patients with NICM and LVEF > 35% undergoing CA for monomorphic VT, procedural noninducibility consistently predicts favourable arrhythmic outcomes. Non-circumferential mid-myocardial LGE with septal involvement and persistent inducibility after ablation identify higher-risk patients. Higher LVEF may confer additional prognostic benefit, and mortality after successful ablation was generally low. However, these findings are derived from limited retrospective data, and prospective validation in larger cohorts is warranted to further inform clinical decision-making in this setting.

## Ethics Statement

The research reported in this paper adhered to the Preferred Reporting Items of Systematic Reviews and Meta-Analyses (PRISMA) statement.

## Patient Consent

The authors confirm that patient consent is not applicable to this article. As this study is a systematic review of published literature, institutional review board approval and informed consent were not required.

## Funding Sources

The authors have no funding sources to declare.

## Disclosures

C.J. was supported by the BMFTR in the framework of the BMFTR Advanced Clinician Scientist Programme UMEA; 01EO2104. K.V. reports receiving research/educational grants from Abbott, Medtronic, and Biosense Webster (all paid to institution); consulting for Medtronic, Abbott, Biosense Webster, Boston Scientific, and Philips (all fees paid to institution). The other authors have no conflicts of interest to disclose.
