# Supplementary material for: Ventricular Tachycardia Recurrence After Catheter Ablation in Nonischemic Cardiomyopathy with Mildly to Moderately Reduced Ejection Fraction
Source: CJC Open. 2026 May 1;8(8):958–67. doi: 10.1016/j.cjco.2026.04.009 (PMC13480278; doi:10.1016/j.cjco.2026.04.009)
Supplement: Supplementary file 1 [file mmc1.pdf]

Supplemental Appendix S1

Complete search strings executed on June 22, 2025.

A. EMBASE

'myocarditis'/exp OR 'amyloidosis'/exp OR 'dilated cardiomyopathy'/exp OR 'sarcoidosis'/exp OR 'nonischemic cardiomyopathy'/exp OR 'familial dilated cardiomyopathy'/exp OR 'arrhythmogenic cardiomyopathy'/exp

AND

'catheter ablation'/exp OR 'ventricular tachycardia ablation'/exp OR 'catheter ablation'

AND

'heart ventricle tachycardia'/exp OR 'Ventricular tachycardia'

B. PUBMED

(cardiomyopathy, dilated[MeSH Terms] OR myocarditis[MeSH Terms] OR sarcoidosis[MeSH Terms] OR amyloidosis[MeSH Terms] OR arrhythmogenic cardiomyopathy[Title/Abstract] OR nonischemic cardiomyopathy[Title/Abstract] OR dilated cardiomyopathy[Title/Abstract] OR myocarditis[Title/Abstract] OR sarcoidosis[Title/Abstract] OR amyloidosis[Title/Abstract] OR arrhythmogenic cardiomyopathy[Title/Abstract] OR nonischemic cardiomyopathy[Title/Abstract] OR non-ischemic cardiomyopathy[Title/Abstract] OR nonischaemic cardiomyopathy[Title/Abstract] OR non-ischaemic cardiomyopathy[Title/Abstract])

AND (catheter ablation[MeSH Terms] OR ablation technique[MeSH Terms] OR catheter ablation[Title/Abstract] OR ablation[Title/Abstract])

AND (ventricular tachycardia[MeSH Terms] OR ventricular tachycardia[Title/Abstract])

C. COCHRANE

([mh "Ventricular Tachycardia"] OR "ventricular tachycardia" OR "ventricular arrhythmia" OR "ventricular premature beats" OR "ventricular ectopy" OR "PVC" OR "VT")

AND ([mh "Ablation Techniques"] OR [mh "Catheter Ablation"] OR "ablation" OR "catheter ablation")

AND ([mh "Cardiomyopathy, Dilated"] OR [mh "Cardiomyopathies"] OR [mh "Myocarditis"] OR [mh "Amyloidosis, Cardiac"] OR [mh "Sarcoidosis"] OR "non-ischemic cardiomyopathy" OR "NICM" OR "idiopathic dilated cardiomyopathy" OR "post-myocarditis" OR "arrhythmogenic cardiomyopathy")

Supplemental table S1: Key procedural characteristics of included studies

Study	Procedure duration	Epicardial access	Complications
Oloriz et al. 2014	NR	64/87 (74%)	10/87 (11%); including 3/87 (3%) in-hospital deaths
Bennett et al. 2022	378.4 ± 159.2 min (near-normal LVEF); 370.2 ± 127.0 min (impaired LVEF)	9/65 (14%)	6/65 (9%)
Kottkamp et al. 1995	NR	0/8 (0%)	0/8 (0%)
Delacretaz et al. 2000	NR	0/NR (0%)	NR
Piers et al. 2013	NR	29/45 (64%)	3/45 (7%); tamponade 1/45 (2%), pericardial bleeding 2/29 epicardial (7%)
Muser et al. 2016	8 (6–10) hours	68/442 (38%)	19/442 (4%)
Vaseghi et al. 2018	295 ± 128 min	278/708 (39%)	46/657 (7%)
Li et al. 2024	NR	8/23 (35%)	2/23 (9%)
Nishimura et al. 2021	NR	NR	NR
Kovacs et al. 2023	NR	NR	NR
Sanzo et al. 2013	NR	7/7 (100%)	0/7 (0%)

Supplemental table S2: Risk of bias using the Robins-I tool.

DOI		First author	Year	D1	D2	D3	D4	D5	D6	D7	Overall
10.1111/j.1540-8159.1995.tb06970.x	Prospective	Kottkamp	1995	Serious	Moderate	Serious	Moderate	Moderate	Low	Moderate	Serious
10.1016/j.hrthm.2021.09.026		Bennett	2022	Moderate	Low	Low	Low	Low	Low	Low	Low
10.1111/j.1540-8167.2000.tb00728.x	Retrospective	Delacretaz	2000	Moderate	Low	Moderate	Moderate	Moderate	Low	Moderate	Moderate
10.1161/CIRCEP.114.001568		Oloriz	2014	Moderate	Low	Moderate	Moderate	Moderate	Low	Moderate	Moderate
10.1161/CIRCEP.113.000089		Piers	2013	Moderate	Low	Moderate	Moderate	Moderate	Low	Moderate	Moderate
10.1161/CIRCEP.116.004333		Muser	2016	Moderate	Low	Low	Moderate	Serious	Low	Moderate	Serious
10.1016/j.jacasi.2024.08.018		Li	2024	Serious	Low	Moderate	Moderate	Moderate	Low	Moderate	Serious
10.1016/j.hrthm.2019.04.015		Castro	2019	Moderate	Low	Low	Moderate	Moderate	Low	Moderate	Moderate
10.1016/j.jacep.2018.05.007		Vaseghi	2018	Serious	Low	Low	Moderate	Moderate	Low	Moderate	Serious
10.1111/jce.16039		Kovacs	2023	Moderate	Low	Moderate	Moderate	Moderate	Low	Moderate	Moderate
10.1016/j.hrthm.2020.12.003		Nishimura	2021	Moderate	Low	Low	Moderate	Moderate	Low	Moderate	Moderate

D1: Risk of bias due to confounding
D2: Risk of bias in classification of interventions
D3: Risk of bias due of selection of participants into the study
D4: Risk of bias due to deviations from intended interventions
D5: Risk of bias due to missing data
D6: Risk of bias arising from measurement of the outcome
D7: Risk of bias in selection of the reported results
